# High density linkage map construction and QTL mapping for runner production in allo-octoploid strawberry *Fragaria* × *ananassa* based on ddRAD-seq derived SNPs

**DOI:** 10.1038/s41598-019-39808-9

**Published:** 2019-03-01

**Authors:** Mohammad Rashed Hossain, Sathishkumar Natarajan, Hoy-Taek Kim, Denison Michael Immanuel Jesse, Cheol-Gyu Lee, Jong-In Park, Ill-Sup Nou

**Affiliations:** 1 Department of Horticulture, Suncheon National University, 255 Jungang-ro, Suncheon, Jeonnam 57922 South Korea; 2 Department of Genetics and Plant Breeding, Bangladesh Agricultural University, Mymensingh, 2202 Bangladesh; 3 University-Industry Cooperation Foundation, Suncheon National University, 255 Jungang-ro, Suncheon, Jeonnam 57922 South Korea; 4 Damyang-gun Agricultural Technology Center, Damyang, 57365 Korea

## Abstract

Recent advances in high-throughput genome sequencing technologies are now making the genetic dissection of the complex genome of cultivated strawberry easier. We sequenced Maehyang (short-day cultivar) × Albion (day-neutral cultivar) crossing populations using double digest restriction-associated DNA (ddRAD) sequencing technique that yielded 978,968 reads, 80.2% of which were aligned to strawberry genome allowing the identification of 13,181 high quality single nucleotide polymorphisms (SNPs). Total 3051 SNPs showed Mendelian segregation in F_1_, of which 1268 were successfully mapped to 46 linkage groups (LG) spanning a total of 2581.57 cM with an average interval genetic distance of 2.22 cM. The LGs were assigned to the 28 chromosomes of *Fragaria* × *ananassa* as determined by positioning the sequence tags on *F. vesca* genome. In addition, seven QTLs namely, *qRU-5D, qRU-3D1, qRU-1D2, qRU-4D, qRU-4C, qRU-5C* and *qRU-2D2* were identified for runner production with LOD value ranging from 3.5–7.24 that explained 22–38% of phenotypic variation. The key candidate genes having putative roles in meristem differentiation for runnering and flowering within these QTL regions were identified. These will enhance our understanding of the vegetative vs sexual reproductive behavior in strawberry and will aid in setting breeding targets for developing perpetual flowering and profuse runnering cultivar.

## Introduction

Cultivated strawberry is favored across the globe for its characteristic appearance, flavor, nutritional qualities and health benefitting anti-oxidative properties^1^ which is evident by the continual increase in the cultivation and production of the crop in the last two decades (FAOSTAT 2018). Moreover, strawberry is an interesting model crop due to its unique reproductive dynamics, having intricate relationship between sexual reproduction *via* flowering and asexual reproduction *via* runnering, which is greatly modulated by environmental cues^2–5^. Strawberry cultivars were characterized as seasonal flowering (also known as short-day, June bearing or non-remontant) or perpetual flowering (also known as day-neutral, long-day, everbearing, rebloomer or remontant) based on the flowering response that heavily depends on photoperiod and temperature^6,7^. In seasonal flowering (SF) genotypes, flowering is initiated by short-day lengths (<14 h) and low temperatures (<15 °C) of autumn which remains dormant during winter and emerges in spring, leading to a single harvest season in summer. The perpetual flowering (PF) genotypes, on the other hand, can initiate flowers at any photoperiod with a moderate range (15–21 °C) of temperature, leading to an extended harvest season from spring until late autumn^7–9^. In SF genotypes, the period of flowering is followed by runnering when primary stolons emerge from the basal axillary buds that elongate and give rise to new clonal plants at varyingly spaced nodes. Whereas, in PF genotypes, these sexual and asexual reproduction period overlaps in summer^4,5,9^. Evidences from both wild and cultivated strawberry show that flowering and runnering are genetically distinct but mutually exclusive processes as the predominance of one over the other is intricately related with genetic and environmental factors^3–5,7^. Longer days and higher temperatures promote runnering in SF genotypes, which in short and cooler days produce branch crowns that are differentiated to inflorescence and hence, increases flowering and crop productivity^5,10,11^. Higher temperatures also increase runnering in PF genotypes, however, the effect of photoperiod were found to be variable by different experiments^3,12–14^. PF genotypes are, in general, preferred for an extended commercial harvest season of fresh berries. However, the poor runnering habit of the PF genotypes compared to SF genotypes hinders their propagation, making it one of the prioritized breeding targets which require wider genetic understanding of the trait^3,15–17^.

The studies on the diploid strawberry identified that the PF and RU trait are controlled by different alleles^18^, while in cultivated strawberry, common genetic control with opposing effect has been reported^3^. Fewer studies reported single dominant gene^19–21^, while many studies favored the polygenic control of the PF trait in cultivated strawberry^17,22^. Developing the genetic maps and fine mapping of the loci in the genome will be helpful to genetically disentangle the trait. However, the complex allo-octoploid (2n = 8x = 56) genome of cultivated strawberry, which consists of four relatively similar sub-genomic chromosome sets from diploid donors posed as a constraint in this regard^23–26^. Several studies have constructed linkage maps using different suite of molecular markers such as RAPD, AFLP and SSR, etc.^27–32^ and few of those studies reported QTLs with various additive effects for PF and RU traits in cultivated strawberry^3,4,15,17,33–35^. However, these markers largely suffer from either non-transferability across investigations, insufficient genome-wide coverage, poor density or higher developmental and screening costs^3,15,36^.

Recent release of strawberry virtual reference genome and advances in reduced sequencing technologies such as ddRAD-seq (restriction site associated DNA sequencing), DArT-Seq (Diversity Arrays Technology) and GBS (genotyping by sequencing) offer the potentiality of rapid and cost effective detection of genome wide single nucleotide polymorphisms (SNPs) that can be used in construction of high density linkage maps and fine mapping of the traits of interest^23,26,37^. Such studies have recently been reported in few polyploid species such as wheat, *Brassica napus*, sugarcane, sweet potato and peanut etc.^38,39^. Very recently, the efficacy of DArT^24^, ddRAD-seq^40^ and GBS^41^ technologies have been reported for developing SNP based linkage maps in cultivated strawberry which showed the way for development of linkage maps with increased marker density and increased genomic span. None of these studies, however, attempted to identify QTLs using these SNP based linkage maps. We thus primarily aimed at developing high density and greater genome covering linkage map using ddRAD-seq derived SNPs and in addition, mapping QTLs for runner production in a population raised by crossing parents with contrasting runner producing capability which will be helpful in understanding the genetics of runnering in strawberry.

## Results

### ddRAD-seq based SNP discovery and genotyping

**Figure 1:**
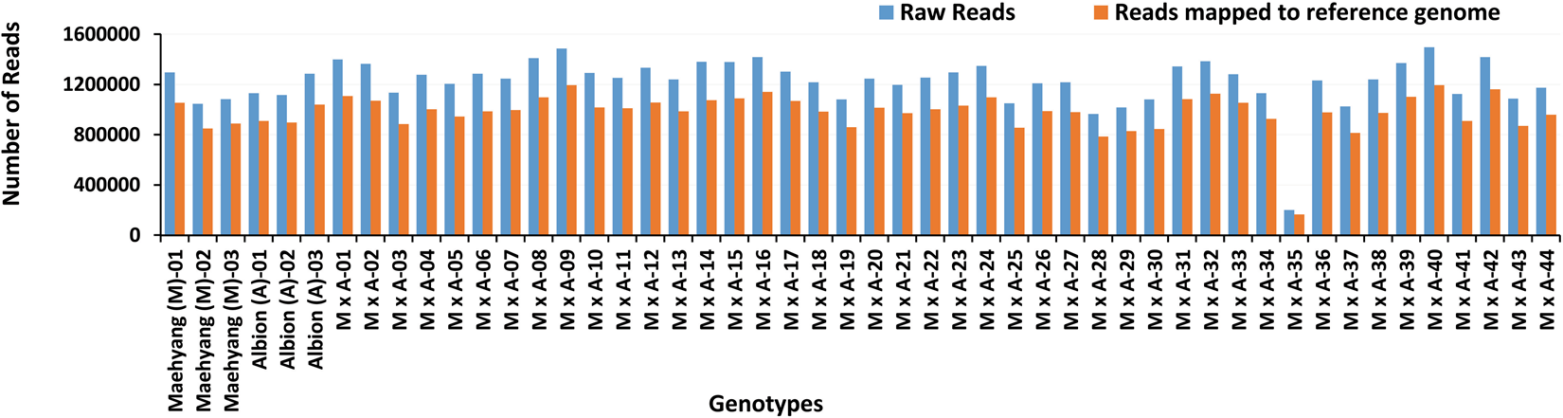
Frequency of mapped and unmapped raw reads obtained from the ddRAD-seq of the strawberry cultivars, Maehyang and Albion, and their F_1_ offspring on the reference genome.

**Figure 2:**
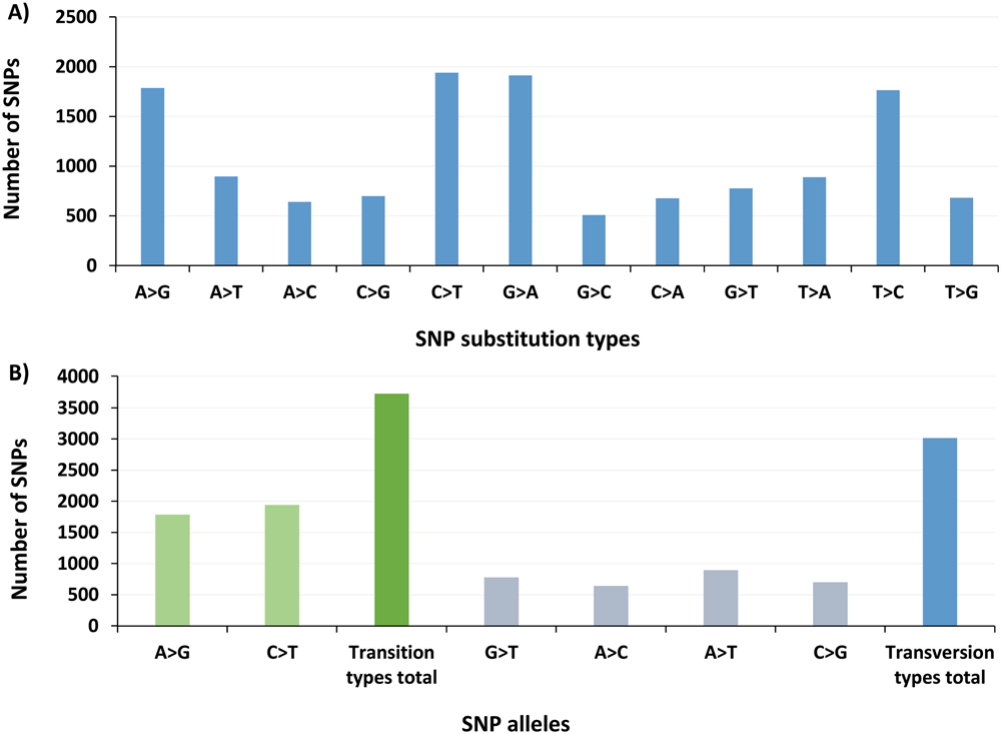
Distribution types (**A**) and transition/transversion ratios (**B**) of SNPs identified from ddRAD-sequencing of strawberry parental and F_1_ population.

The ddRAD (*PstI* and *MspI*) representation libraries, prepared from the genomic DNA of Maehyang × Albion (M × A) populations were successfully sequenced using the Illumina HiSeq platform. The paired-end sequencing of individuals generated a total of 61,065,374 reads (10 GB sequence data). On average, 978,968 high quality reads per sample (80.2% of total reads) were aligned against the *Fragaria* × *ananassa* genome version *FAN_r1.1* (Fig. 1). The statistics of raw reads, cleaned reads, reads mapped to the reference genome and alignment ratio for individual accessions were summarized in Supplementary Table S1. The mapped reads were further investigated to select a total of 13,181 high quality SNPs based on strict SNP filtration criteria (SNP quality score ≥ 999, minimum depth = 5, minimum allele frequency = 0.05 and minimum proportion of missing data = 0.5) through VCFTools program. The SNPs were distributed as follows, 678 (5%) C/A, 1914 (14%) G/A, 508 (4%) G/C, 889 (7%) T/A, 1765 (13%) T/C and 683 (5%) T/G (Fig. 2A). Cumulatively, the SNP transitions (A/G or C/T) were higher (3728) compared to transversions (G/T, A/C, A/T or C/G) (3016) and the transition/tranversion ratio was 1.23 (Fig. 2B). Overall, the highest SNP frequencies were observed for transition C/T (1941) followed by A/G (1787). In addition, transition/tranversion (TS/TV) ratio of >0.5 was used for calculating divergence and restructuring the phylogenetic tree^42^. The high quality SNPs were annotated using SnpEff tool (version 4.4) and the generated database was used to annotate possible SNPs based on their genomic, exonic and functional classes. A total of 6,094 SNPs (34.7%) were exonic while 1,844 (10.5%) and 3,245 (18.7%) were intronic and intergenic SNPs, respectively (Supplementary Fig. S1). The lowest number of SNPs (1.9%) were located in the 5′UTR region followed by 4.3% (759) located in 3′UTR region.

### Construction of high density linkage map

**Figure 3:**
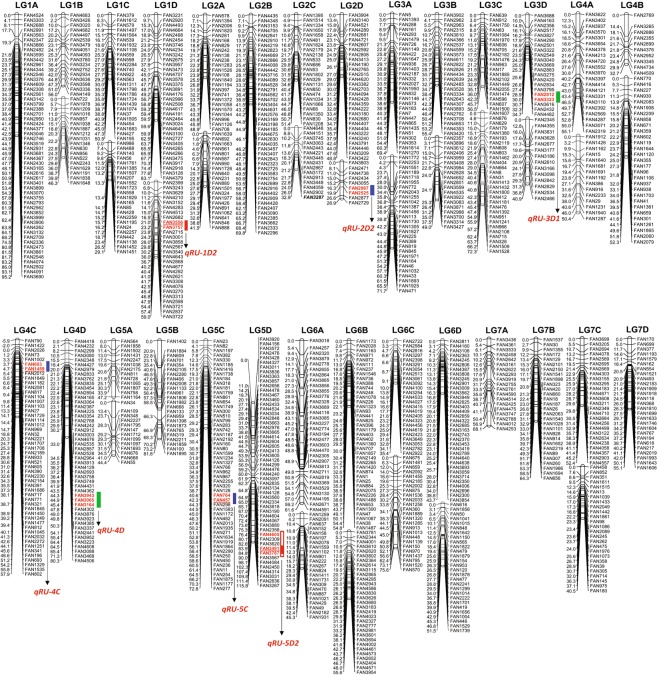
A SNP-based linkage map of a *Fragaria* × *ananassa* mapping population derived from the progeny of the cross ‘Maehynag × Albion’ comprising of 1292 SNP markers. The genetic distances are given in centi-Morgans (cM) in left. Green, blue and red bars indicate QTLs detected by CIM method, multi-locus GWAS methods (mrMLM, FASTmrMLM, pLARmEB and ISIS EM-BLASSO) and all of these methods, respectively. Underlined marker indicate the central marker within the QTLs that are identified by multi-locus GWAS methods.

Among the 13,181 high quality SNPs identified, a total of 4738 were selected based on the presence of heterozygous loci either in maternal or paternal or both parents for linkage analysis. A total of 1687 markers showed distortion from Mendelian segregation. Among the 3051 markers showing significant Mendelian segregation, 1064 pairs (consisting of 932 unique markers) showed exactly identical (Supplementary Table S2) and 1010 pairs (consisting of 961 unique markers) showed highly similar (Supplementary Table S3) patterns of segregation. Finally, 1268 SNP markers were mapped to the 46 linkage groups spanning a genetic distance of 2581.57 cM along the length of the 7 diploid *F. vesca* chromosomes representing all 28 chromosomes of octoploid *Fragaria* × *ananassa* (Table 1; Fig. 3). The remaining markers that were not included were either not linked to any of the recognized LGs, were mapped in small groups or showed conflicting segregation pattern with other markers of the same linkage group at the selected LOD threshold. The lengths of linkage groups ranged from 28.93 cM (LG2D2) to 115.84.1 cM (LG5D) with an average length of 56.12 cM. The map resolution corresponded to an average marker density of one marker every 0.48 cM with the longest marker interval of 20.94 cM observed in LG5B. The physical mapping of the SNP markers on the *F. vesca* genome (v4.0.a1) sequences revealed a total coverage of 76.8% (184.33 Mb) of the diploid genome (240 Mb) which represents one of the four sub-genomes of the cultivated octoploid *Fragaria* × *ananassa*. The details of linkage group-wise markers, marker resolutions, genetic distance on *Fragaria* × *ananassa* and corresponding physical position on diploid *F. vesca* are shown in Table 1 and Supplementary Table S4 and the corresponding gene IDs, the SNP positions and genotypes are shown in Supplementary Table S5. High degree of collinearity between the genetic distances of the mapped SNP markers of each linkage group and their corresponding physical position on the *F. vesca* chromosomes were observed for most of the LGs except for few shorter and less dense LGs such as LG1B2, LG2D2, LG4B1, LG5A1 and LG5A2 (Supplementary Fig. S2).

**Table 1:** Summary statistics of the linkage groups constructed using the population arising from the cross ‘Maehyang × Albion’ along with their physical distances on the *F. vesca*_v4.0.a1 genome sequences.

SL	Linkage group	Length (cM)	First and last mapped marker	Total no. of mapped markers	Average interval (cM/locus)	Longest gap (cM)	*F. vesca* chromo-some	LG physical start (bp)^a^	LG physical end (bp)^b^	Physical Span (bp)^c^
1	LG1A	95.25	FAN4524-FAN3690	54	1.76	9.64	Fvb1	112529	16227481	16114952
2	LG1B1	55.99	FAN4683-FAN4149	17	3.29	7.78	Fvb1	144009	4479442	4335433
3	LG1B2	46.33	FAN722-FAN1648	18	2.57	6.47	Fvb1	1383360	5403172	4019812
4	LG1C1	50.53	FAN379-FAN195	26	1.94	8.31	Fvb1	6183315	10129223	3945908
5	LG1C2	29.13	FAN986-FAN1451	23	1.27	7.36	Fvb1	7969400	20650547	12681147
6	LG1D1	83.11	FAN3231-FAN4379	34	2.44	9.88	Fvb1	3921057	18970557	15049500
7	LG1D2	58.98	FAN2399-FAN3722	28	2.11	6.76	Fvb1	7438667	19123178	11684511
8	LG2A1	48.88	FAN578-FAN708	24	2.04	7.14	Fvb4	804175	11904624	11100449
9	LG2A2	41.55	FAN1639-FAN888	20	2.08	6.38	Fvb4	425782	13276588	12850806
10	LG2B	69.78	FAN4435-FAN2296	45	1.55	7.09	Fvb2	267888	28873870	28605982
11	LG2C1	51.38	FAN1395-FAN1208	23	2.23	7.86	Fvb2	5674815	29167427	23492612
12	LG2C2	32.94	FAN4648-FAN3287	15	2.20	8.59	Fvb2	15750516	19803671	4053155
13	LG2D1	48.82	FAN3904-FAN3422	30	1.63	13.42	Fvb2	24250389	29150635	4900246
14	LG2D2	28.93	FAN3803-FAN2729	9	3.21	8.09	Fvb2	21804777	26832106	5027329
15	LG3A1	69.38	FAN5-FAN1270	29	2.39	11.75	Fvb3	26607	12531139	12504532
16	LG3A2	71.72	FAN1772-FAN471	28	2.56	19.38	Fvb3	2288930	37706799	35417869
17	LG3B1	55.36	FAN3902-FAN3420	25	2.21	4.12	Fvb3	3610045	14574195	10964150
18	LG3B2	48.96	FAN2357-FAN3427	19	2.58	11.49	Fvb3	8122733	13333825	5211092
19	LG3C1	56.48	FAN639-FAN1500	20	2.82	10.76	Fvb3	12328117	36511410	24183293
20	LG3C2	72.33	FAN1607-FAN1528	29	2.49	6.38	Fvb3	17089842	38167538	21077696
21	LG3D1	34.68	FAN3688-FAN4482	21	1.65	10.02	Fvb3	33191108	37766726	4575618
22	LG3D2	40.92	FAN2345-FAN2466	14	2.92	12.77	Fvb3	30017669	38165007	8147338
23	LG4A1	55.10	FAN3622-FAN2319	21	2.62	12.32	Fvb4	1372325	19654576	18282251
24	LG4A2	45.95	FAN1912-FAN1940	15	3.28	6.75	Fvb4	572887	22338700	21765813
25	LG4B1	32.73	FAN2769-FAN4509	10	3.27	14.41	Fvb4	2824575	4495688	1671113
26	LG4B2	52.26	FAN770-FAN2079	29	1.80	5.34	Fvb4	6686516	27802611	21116095
27	LG4C	57.91	FAN2026-FAN802	51	1.14	3.89	Fvb4	344401	33602364	33257963
28	LG4D	80.28	FAN4418-FAN4506	66	1.22	8.99	Fvb4	1119936	33498276	32378340
29	LG5A1	47.20	FAN564-FAN1164	13	3.63	13.32	Fvb5	319932	2289061	1969129
30	LG5A2	44.43	FAN34-FAN55	13	3.42	13.37	Fvb5	7581891	9861778	2279887
31	LG5B	81.79	FAN1402-FAN1895	24	3.41	20.94	Fvb5	3768759	18410022	14641263
32	LG5C	72.80	FAN23-FAN277	54	1.35	4.47	Fvb5	130730	15080365	14949635
33	LG5D	115.84	FAN3920-FAN3267	73	1.59	11.00	Fvb5	21228806	27416727	6187921
34	LG6A1	61.62	FAN3018-FAN4591	38	1.62	6.24	Fvb6	2083928	7251058	5167130
35	LG6A2	45.34	FAN1259-FAN1931	23	1.97	10.04	Fvb6	70354	11645796	11575442
36	LG6B1	54.78	FAN1173-FAN1487	41	1.34	5.05	Fvb6	1660733	10248262	8587529
37	LG6B2	55.64	FAN3781-FAN3954	31	1.79	7.67	Fvb6	5864047	15255602	9391555
38	LG6C1	35.35	FAN2722-FAN2922	25	1.41	3.31	Fvb6	10630696	20387841	9757145
39	LG6C2	75.55	FAN675-FAN570	25	3.02	10.68	Fvb6	3657849	27953227	24295378
40	LG6D1	43.69	FAN3811-FAN3854	35	1.25	4.88	Fvb6	30923227	39197465	8274238
41	LG6D2	51.45	FAN616-FAN1739	28	1.84	6.17	Fvb6	15955826	37293159	21337333
42	LG7A	68.14	FAN3436-FAN4253	19	3.59	10.79	Fvb7	1700487	8698128	6997641
43	LG7B	64.28	FAN1537-FAN656	29	2.22	7.15	Fvb7	7860566	22021634	14161068
44	LG7C1	52.37	FAN3699-FAN4557	26	2.01	7.16	Fvb7	5168144	20476274	15308130
45	LG7C2	40.51	FAN458-FAN180	26	1.56	7.56	Fvb7	17500684	24064058	6563374
46	LG7D	55.15	FAN170-FAN1606	27	2.04	8.11	Fvb7	16557672	24078363	7520691
**Total**	n/a	**2581.57**	n/a	**1268**	**2.22**	n/a	n/a	n/a	n/a	**184.33 Mb** ^**d**^

^a,b^The overall physical start and end positions of all markers of one linkage group (that are usually mapped to a particular *F. vesca* chromosome) in the *F. vesca*_v4.0.a1 genome.

^c^The distance between the LG physical start and LG physical end that indicates the overall physical span of all the markers of one linkage group in a particular chromosome of the *F. vesca*_v4.0.a1 genome.

^d^The sum of chromosome-wise overall physical distances (span between the first and last mapped markers in a particular *F. vesca* chromosome) covered by the markers in all seven *F. vesca* (v4.0.a1 genome) chromosomes.

### Phenotypic evaluation

**Figure 4:**
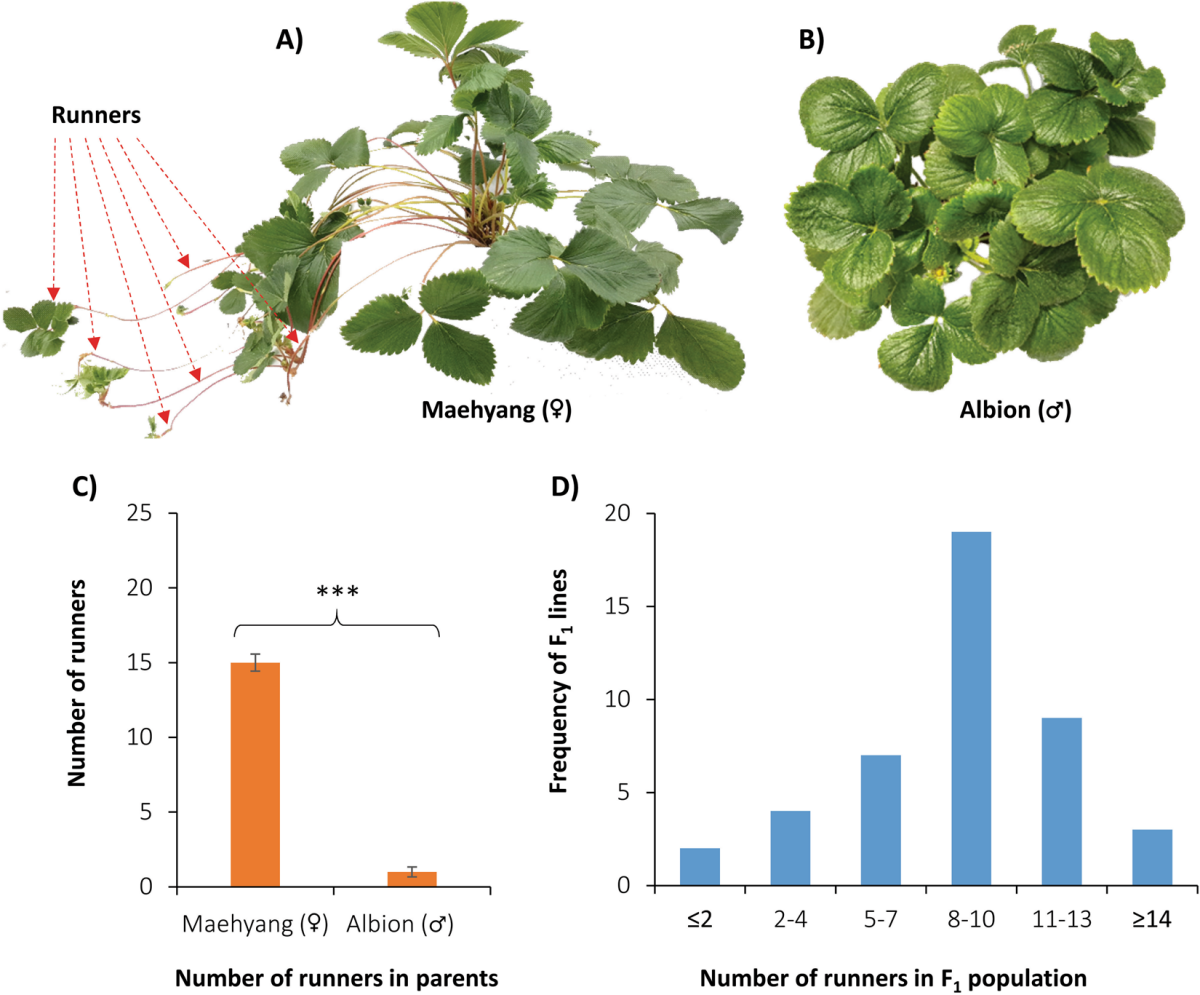
Runnering behavior of the profuse runner producing and seasonal flowering parent, Maehyang (**A**) and poor runner producing and perpetual flowering parent, Albion (**B**); and the frequency distribution of the total number of runners in parents (**C**) and in their F_1_ population (**D**). For figure C, data presented as mean ± SE (n = 5) and asterisks (***) represent significant differences between the two parental lines for total number of runners at the end of the growing season at P < 0.001 by Student’s *T*-test.

The two cultivars exhibited significant variation (p < 0.001) in terms of number of runner production in one growing season (Fig. 4A–C). The seasonal flowering cultivar ‘Maehyang’ produced 15 runners while the perpetual flowering cultivar produced only one runner during the entire growing season (Fig. 4C). In the segregating F_1_ population derived from crossing these two contrasting cultivars, continuous variation was observed for total number of runners (Fig. 4D). The minimum (0) and the maximum (15) number of runners were produced by one and two F_1_ lines, respectively while a moderate 8–10 runners were produced by 19 F_1_ lines (Fig. 4D).

### Identification of QTLs

Using the composite interval mapping and several multi-locus GWAS methods, we identified five negative effect QTLs namely, *qRU-5D, qRU-3D1, qRU-1D2, qRU-4D* and *qRU-2D2* located on linkage groups LG5D, LG3D1, LG1D2, LG4D and LG2D2, respectively and two positive effect QTLs namely, *qRU-4C* and *qRU-5C* located on linkage groups LG5C and LG5C, respectively related to the number of runners in the F_1_ population raised from crossing Maehyang × Albion (Table 2; Fig. 3). The QTL *qRU-5D* identified on LG5D had the highest LOD score of 7.24 (p < 0.05) and explained the highest proportion (38%) of phenotypic variation (R^2^) among the identified QTLs. This QTL was associated with the flanking markers, FAN4605 and FAN3787 (83.88–84.81 cM) on the linkage group LG5D. The next highest LOD (7.15) was observed for the QTL *qRU-3D1* (additive effect = −8.14; R^2^ = 0.36; flanking markers = FAN2812-FAN3323).

**Table 2:** Map positions and genetic effects of QTLs detected for the number of runner (RU) in F_1_ population derived from crossing octoploid strawberry cultivars Maehyang and Albion.

QTL name	Linkage Group	Marker	Genetic distance (cM)^a^	*Fragaria* × *ananassa* genome ID	*F. vesca* Physical position (bp)	Threshold LOD^b^	LOD^c^	Additive Effect	R^2d^
qRU-5D**	LG5D	FAN4605-FAN3787	83.88–84.81	FAN_iscf00235992.1-FAN_iscf00019356.1	Fvb5:23272711-23422418	2.7	7.24	−9.79	0.38
*qRU-3D1*	LG3D1	FAN2812-FAN3323	28.9–30.04	FAN_iscf00310724.1-FAN_iscf00161563.1	Fvb3:37021445-37232303	2.9	7.15	−8.14	0.36
qRU-1D2**	LG1D2	FAN3556-FAN3757	20.99–23.23	FAN_iscf00315119.1-FAN_iscf00003334.1	Fvb1:9879715-9988189	2.9	5.54	−8.54	0.37
*qRU-4D*	LG4D	FAN3943-FAN3164	43.51–45.28	FAN_iscf00171554.1-FAN_iscf00369931.1	Fvb4:23126333-24533627	2.9	3.5	−5.12	0.22
qRU-4C*	LG4C	FAN603-FAN1459	3.74–4.73	FAN_iscf00089616.1-FAN_iscf00083346.1	Fvb2:16350821-17032825	2.8	4.11	3.663	0.32
qRU-5C*	LG4C	FAN764-FAN457	40.58–42.31	FAN_iscf00280849.1-FAN_iscf00206802.1	Fvb5:8718088- 10445687	2.7	3.97	3.182	0.24
qRU-2D2*	LG2D2	FAN2967-FAN2525	16.31–19.90	FAN_iscf00134314.1-FAN_iscf00342145.1	Fvb2:23649400-23980136	2.8	3.96	−4.37	0.28

No asterisk, * and ** indicate QTLs detected by composite interval mapping (CIM), multi-locus GWAS methods (mrMLM, FASTmrMLM, pLARmEB and ISIS EM-BLASSO method) and all of these techniques, respectively.

^a^The distance of the QTL in cM (expressed in Kosambi) from the top of the linkage group.

^b^The significant threshold logarithm of the odds (LOD) score calculated by the composite interval mapping using Kosambi’s map function with 1000 permutation (p ≤ 0.05).

^c^The peak LOD score.

^d^The percentage of phenotypic variance explained by the QTLs as determined by QTL Cartographer version 2·5 (NC, USA).

### Candidate genes within the QTL regions

Altogether 587 genes were found within the seven QTL regions, with a maximum of 196 genes within QTL *qRU-5C* and minimum of 19 genes within *QTL qRU-5D* and *qRU-1D2* each (Supplementary Table S6). Among these, the key candidate genes having putative roles in the vegetative vs reproductive differentiation of shoot apical meristems and regulation of flowering are listed in supplementary Table S7. For example, WUSCHEL related homeobox 1 (FvH4_5g17270) and AGAMOUS-like 71 (FvH4_4g20680) within QTL *qRU-4D*, CLAVATA3/ESR (CLE)-related protein coding gene FvH4_2g19210 within *qRU-4C* and Subtilisin-like protease (FvH4_1g17150, FvH4_1g17160 and FvH4_1g17170) within qRU-1D2 may have roles in the CLAVATA-WUSCHEL signaling pathway that specifies meristematic stem cell fate during differential of shoot and floral buds. Several genes encoding cytokinin dehydrogenase 7 (FvH4_2g30990, FvH4_2g31000 and FvH4_2g31010), auxin response factor 17-like (FvH4_2g19860), auxin efflux carrier family protein (FvH4_5g173100), NAC domain-containing protein 86 (FvH4_5g18130) and homeobox protein knotted-1-like gene *KNOXI* (FvH4_5g17670) could be involved in cytokinin-auxin mediated meristem differentiation. In addition, a total of 12 tetratricopeptide repeat (TPR)-like superfamily protein genes such as FvH4_5g32370; FvH4_5g32380 and FvH4_2g30790 etc. are found within all but *qRU-3D1* QTLs which may have roles in the maintenance of meristem cell organization. Two phytochrome related genes, FvH4_4g19750 and FvH4_2g19790 identified within QTLs *qRU-4D* and *qRU-4C*, respectively, could be involved in the photoperiodic control of flowering. In addition, six genes namely, FvH4_4g20210.1 encoding ‘flowering time control protein FCA-like isoform X1’ within the QTL *qRU-4D*, FvH4_2g19520.1 encoding ‘zinc finger protein KNUCKLES-like protein’ within the QTL *qRU-4C*, FvH4_2g30770.1 encoding ‘flowering-promoting factor 1-like protein (*FPF1*)’ within the QTL *qRU-2D2*, FvH4_5g17130 encoding ‘phosphatidylethanolamine-binding protein (*PEBP*)- mother of *FT (FLOWERING LOCUS T)* and *TF1 (TERMINAL FLOWER 1)*’ within *qRU-5C* and FvH4_5g18150 and FvH4_5g18160 encoding ‘protein argonaute 1 (*AGO1*) within *qRU-5C* may have potential roles in control of flowering time. Three translation elongation factor genes *EF1B* (FvH4_4g20660), *EIF3A* (FvH4_4g19950) and *EF1A* (FvH4_5g17000) could be involved in meristem stability and organogenesis.

## Discussion

The intricate interplay between vegetative and sexual reproduction with the regards to the environmental cues have both biological and economic significance. This makes strawberry an important model system to study flowering behavior. Runner and flower arise from different meristem crowns (basal and terminal, respectively) based on competitive investment of resources triggered mainly by environmental factors^4,7,13,43^. Genetics of these two mutually exclusive and antagonistic processes of wild and cultivated strawberry has long been investigated and several hypotheses, mostly favoring polygenic over monogenic control has been put forwarded by previous studies^2,5,15,18,19,44^. Inheritance studies and mapping of the traits of interest at molecular level necessitated the construction of linkage maps. Here, we report the construction of a linkage map and identification of QTLs for number of runners based on the SNPs detected in the F_1_ progeny developed from the cross of profuse and poor runnering cultivars, Maehyang and Albion, respectively by aligning the ddRAD-seq reads against strawberry genome (FAN_r1.1).

History of construction of LGs in strawberry followed distinct phases, starting from the initial low density and non-transferrable marker based LGs^17,27^ followed by the use of a comprehensive suite of sequence characterized and transferrable markers^21,28,32^ which were further updated upon the release of the diploid *F. vesca* genome and octoploid *Fragaria* × *ananassa* draft genome^29,36,45^ and finally the development of high-throughput SNP based LGs^24,40,41,46^. The first genetic linkage map in octoploid strawberry were constructed on the full-sib progeny of ‘Capitola’ × ‘CF1116’, where 235 and 280 single dose restriction fragment (SDRF) markers were mapped on the 43 co-segregating groups spanning a total of 1604 and 1494 cM in female and male parents, respectively^27^. Rousseau-Gueutin *et al*.^32^ included an additional 306 SSR and 5 STS and SCAR markers in the original AFLP marker set of Lerceteau-Köhler *et al*.^27^ and constructed 28 and 26 LGs spanning 2582 and 2165 cM in female and male parents, respectively in the extended ‘Capitola × ‘CF1116’ population. Weebadde *et al*.^17^ further identified 43 linkage groups using 429 AFLP markers on a population of the cross ‘Tribute’ (day-neutral) × ‘Honeoye’ (short-day). Using 71 RAPD markers in a population of 199 F_1_ plants of the cross ‘Ever Berry’ (Japanese everbearing cultivar) and ‘Toyonoka’ (Japanese seasonal flowering cultivar), Sugimoto *et al*.^21^ identified a linkage group of 39.7 cM that harbor the everbearing gene in strawberry. Using a combination of 315 AFLP, RAPD, SSR and gene specific markers on the 174 F_1_ progeny of the cross ‘Redgauntlet’ × ‘Hapil’, Sargent *et al*.^28^ constructed 37 and 32 LGs spanning 1440.7 and 1675.3 cM in the male and female parents, respectively. The observation of high collinearity between the diploid and octoploid strawberry linkage maps^28,32^ prompted others^29,36,45^ to further populate the linkage maps of octoploid strawberry. More markers were designed from the freshly released diploid strawberry genome in 2011^26^ which enabled to saturate the gaps and increase the genomic coverage of previous linkage maps in different populations.

The release of octoploid reference genome^23^ enabled the development of linkage groups based on SNPs detected by various techniques^24,25,40,41,46^. Davik *et al*.^40^ first demonstrated the effectiveness of ddRAD-seq derived SNPs by constructing a 1518.5 cM robust, high resolution linkage map consisting of 31 linkage groups from a population of Sonata × Babette. We have identified 46 linkage group fragments that spanned a higher genetic distance (2581.57 cM) with an average marker space of only 0.48 cM in M × A crossing population. Mapping of the marker associated sequence tags of each of the LGs to the updated *F. vesca* genome assembly (v4.0.a1) revealed a reasonable genome-wide coverage (76.8% of the total 240 Mb) of the diploid wild relative’s genome. The markers of a particular LG were usually mapped to a single *F. vesca* chromosome. However, for some LGs, few markers (a total of 185 out of 1268 mapped markers with an average of 4.02 marker per LG) were mapped to different chromosomes (Supplementary Table S4). The highest 20 markers (out of 73) of LG5D were mapped to a different chromosome followed by LG4C (18 out of 51). This is in agreement with the report of highly conserved macro-synteny between diploid and octoploid strawberry with few inter-chromosome rearrangements^25,29,32^. These high degree of collinearity further indicates the reliability of the constructed linkage map. However, it is noteworthy that 41% (1268) of the total markers (3051) which showed significant Mendelian segregation ratio were assigned to LGs and there are five LGs with less than 15 markers and two LGs with a longest gap of ~20 cM. This could be attributed to the stringent analysis parameters such as a high LOD (>5) aiming at constructing a representative linkage map by avoiding spurious linkage that can facilitate accurate detection of QTL.

Using the SNP marker based linkage group, we are the first to report QTL in *Fragaria* × *ananassa*. Besides the composite interval mapping, we used multi-locus GWAS methods such as mrMLM, FASTmrMLM, pLARmEB and ISIS EM-BLASSO which is shown to detect minor effect QTLs^47–50^. Altogether, seven QTLs, two with relatively high LOD scores (>7.0) were identified which further strengthens the polygenic control of runnering hypothesis^8,17^. Gaston *et al*.^3^ demonstrated common genetic control of flowering and runnering by a major dominant locus (*FaPFRU*) which positively influences flowering and exerts a negative effect on runnering. Of the seven QTLs identified in this study, five were negative effect QTLs. Using SSR marker derived LGs, a single major QTL for runnering and perpetual flowering reported by Castro *et al*.^15^ was further confirmed by Sooriyapathirana *et al*.^33^ using an extended population. Using three separate Japanese population, Honjo *et al*.^35^ mapped the PF trait to similar region of previously detected QTL regions.

The breeding industry requires both perpetual flowering and profuse runnering traits but the PF genotypes are poor runner producers. Several putative candidates for PF have already been reported in wild and cultivated strawberry such as *TFL1, KSN, FT1, FT2, FT3, FvSOC1* and *TCP7*^34,51–55^. However, their exact roles in the intricate interplay between meristem differentiation leading to inflorescence or runners are yet to be fully established. In this study, we have mined the genes within the QTL regions and identified several key candidate genes having putative roles in CLAVATA-WUSCHEL signaling, cytokinin–auxin mediated meristem differentiation, directional cell division, meristem stability and organogenesis, gravitropism, vernalization and photoperiodic regulation of flowering etc. that ultimately determines the stem cell fate towards shoot or floral meristem differentiation^56–59^ (Table S7). Functional characterization of these genes will enhance our understanding of the complex interplay between vegetative vs sexual propagation in strawberry. In addition, the sequence polymorphisms such as the peak QTL SNP markers identified in this study could be used for marker assisted breeding (Table 2). Very recently, the gene *FveRGA1* (gene06210 in *F. vesca*_v1.0) encoding a putative DELLA protein GAIP-B has been shown to play a role in stolon production in diploid strawberry^60^. It’s corresponding homologue located in FAN_iscf00392249 (Fvb4:32063138..32064973) of *Fragaria* × *ananassa* did not inhabit any SNP in our study population. However, one SNP was found in their closest genetic locus FAN_iscf00207522.1 (Fvb4: 32318115..32316672) in the linkage group LG4D at 19.19 cM apart from our qTL *qRU-4D*.

In conclusion, we report a linkage map with higher density and greater genome-wide span using the ddRAD-seq derived SNPs from the progeny of two strawberry cultivars with contrasting flowering and runnering attributes. In addition, we have also identified QTLs for total number of runners. This high-throughput genotyping based linkage map will serve as a reference for precise sequence scaffold anchoring and orientations in this species and the approach can be replicated in other genetically complicated species. The candidate genes identified within the QTLs, upon functional validation, can be targeted for biotechnological manipulations to develop genotypes with desirable flowering and runnering habit. Besides, the large number of SNPs will supply abundant choices of transferrable markers for future genetic studies and will assist in identifying QTLs, causal genes and linked markers for agronomically important traits which in turn will accelerate strawberry genetic improvement programs *via* molecular breeding.

## Materials and Methods

### Plant materials and phenotyping

The experimental population included 44 F_1_ hybrid seedlings raised by crossing the seasonal flowering (SF) short-day cultivar Maehyang (♀) and the perpetual flowering day-neutral cultivar Albion (♂) (M × A). The F_1_ seeds were germinated in growth chamber before being transplanted to large rectangular pots in the farm facility of Damyang-gun Agricultural Technology Center, South Korea specialized for strawberry cultivation. The young leaves from the F_1_ progenies and three sets of both the parents were chosen for further procedure. The cultivars Maehyang and Albion are economically important and harbor several other contrasting growth and fruit qualitative traits in them. Total number of runners were recorded by counting and removing the newly emerged primary stolons twice a week during one growing season under natural temperature and photoperiodic conditions.

### DNA extraction

Newly emerged fresh leaves from the parents and F_1_ plants were disrupted in TissueLyser II (Qiagen, CA, USA). The DNA extracted using DNeasy Plant Mini Kit (Qiagen) following manufacturer’s instructions. The quality of DNA was assessed in agarose gel (1.2%) electrophoresis. The concentration and purity of the extracted DNA were evaluated using Nanodrop-2000 (Nanodrop Technologies, Wilmington, DE, USA).

### Double digest restriction site associated DNA (ddRAD) sequencing

Genomic DNA of each strawberry accession was double-digested with the restriction enzymes, *PstI* and *MspI* to prepare the ddRAD-seq libraries. The unique barcode of 8 nucleotide base pairs with Illumina adaptor was assigned to each strawberry accession for tracing the samples (Supplementary Table S1). The adaptor-ligated DNA amplicons were pooled and DNA fragments of 300–900 base pair length were separated with BluePippin (Sage Science, Beverly, MA, USA). The constructed ddRAD-seq libraries were sequenced on HiSeq platform (Illumina, USA) using 93 base paired-end (PE) mode as described by Shirasawa *et al*.^38^.

### Sequence data analysis and SNP detection

Primary data processing of ddRAD-seq was performed as per the procedures described in Shirasawa *et al*.^38^ with minor modifications. The sequencing data of strawberry accessions were examined for their quality using FastQC tool (http://www.bioinformatics.babraham.ac.uk/projects/fastqc/). The low-quality sequences and adaptor sequences were trimmed using PRINSEQ (http://prinseq.sourceforge.net)^61^ and fastx_clipper in FASTX-Toolkit (version 0.10.1; http://hannonlab.cshl.edu/fastx_toolkit), respectively. The obtained high quality reads from each accession were then mapped to the genome of *Fragaria* × *ananassa* (FAN_r1.1, http://strawberry-garden.kazusa.or.jp/) as reference using Bowtie 2 tool (version 2.1.0; parameters: -minins 100 -no-mixed)^62^. The resulting sequence alignment/map format (SAM) files were converted into Binary Alignment Map (BAM) files and thereafter, SAMtools (version 0.1.19; parameters: -Duf) was used for sorting, indexing and removal of duplicates^63^. Thereafter, genomic variants (SNPs) were called out for each strawberry accessions against reference genome using mpileup module from SAM tools (parameters: -Duf) and BCF tools (parameters: -vcg). In addition, the produced variant call format (VCF) files including SNP details were further filtered as per procedures described by Shirasawa *et al*., (2017) using VCFtools (version 0.1.11)^64^. Furthermore, missing data were imputed using Beagle4 software package (version 5.0)^65^. The effect of SNP annotations on gene functions were predicted using SnpEff tool (version 4.11)^66^.

### Construction of linkage map

The linkage analysis was performed using the SNP-based markers that were homozygous in one parent and heterozygous in the other parent (segregation type code lm × ll and nn × np in JoinMap version 4.1) and heterozygous in both parents (segregation type code hk × hk). Markers with more than 10% missing data and markers that did not fit significantly with the segregation ratio of 1:1 or 1:2:1 in the progeny as per the chi-square test of goodness of fit (p < 0.05) were excluded from map construction. The regression mapping algorithm and Kosambi’s mapping function with a maximum recombination fraction of 0.45, goodness-of-fit jump threshold of 5 and a ripple value of 1 were used for calculating marker order and the map distances were expressed in centi-Morgans (cM) using JoinMap v4.1 (Kyazma, Wageningen, The Netherlands). Linkage groups with a minimum logarithm of odds (LOD) score limit of 5.0 were visualized in MapChart (version 2.32)^67^. All the linkage groups except the smaller ones (<10 markers) were mapped on the *F. vesca* (genome v4.0.a1) genome. The LGs were named LG1 to LG7 based on the number of *F. vesca* chromosome to which the marker associated sequences of each of the LGs were mapped. The suffixes ‘A’-‘D’ were assigned to the LGs mapped to a particular chromosome based on their sequential physical position throughout the length of that chromosome. If any two small linkage groups were found to be mapped to a particular segment of a *F. vesca* chromosome sequentially, they were considered as one linkage group.

### QTL identification

QTLs were mapped based on the marker and phenotypic information of the parents and each individual of the mapping population by the composite interval mapping (CIM) analysis with the following parameters (linkage map method = 10, segregation test size = 0.01, linkage test size = 0.35, mapping function = Kosambi, objective function = SAL and step size = 1 cM) using Windows QTL Cartographer (version 2.5_011) program^68^. In addition, QTLs were also identified using several ‘multi-locus mixed linear model’ such as mrMLM, FASTmrMLM, pLARmEB and ISIS EM-BLASSO methods in R program^47–50^. Putative QTLs were declared based on the significant logarithm of odds (LOD) threshold determined by 1000 permutations^69^. The square of the partial correlation coefficient (R^2^) indicates the proportion of phenotypic variation explained by a QTL. The genes along with their putative functions that lie within the QTL regions were identified against *Fragaria vesca* annotated genome version v4.0.a1 using the *Fragaria* × *ananassa* genome IDs corresponding to the flanking markers of the QTLs as blast queries.

## Supplementary information

Supplementary Info

Supplementary Dataset 2

Supplementary Dataset 3

Supplementary Dataset 4

Supplementary Dataset 5

Supplementary Dataset 6

## Data Availability

The ddRAD-seq raw reads generated in this study were deposited into Sequence Read Archive (SRA) under NCBI accession PRJNA478299 accessible from (https://www.ncbi.nlm.nih.gov/sra/PRJNA478299).
