# Supplementary material for: High density linkage map construction and QTL mapping for runner production in allo-octoploid strawberry Fragaria × ananassa based on ddRAD-seq derived SNPs
Source: Sci Rep. 2019 Mar 1;9:3275. doi: 10.1038/s41598-019-39808-9 (PMC6397268; doi:10.1038/s41598-019-39808-9)
Supplement: Supplementary file 1 — Supplementary Info [file 41598_2019_39808_MOESM1_ESM.docx]

**Supplementary Information**

**High density linkage map construction and QTL mapping for runner production in allo-octoploid strawberry *Fragaria* × *ananassa* based on ddRAD-seq derived SNPs**

Mohammad Rashed Hossain^1,2,‡^, Sathishkumar Natarajan^1,‡^, Hoy-Taek Kim^1,3,^*, Denison Michael Immanuel Jesse^1^, Cheol-Gyu Lee^4^, Jong-In Park^1^, Ill-Sup Nou^1,^*

^1^ Department of Horticulture, Suncheon National University, 255 Jungang-ro, Suncheon, Jeonnam 57922, South Korea

^2^ Department of Genetics and Plant Breeding, Bangladesh Agricultural University, Mymensingh 2202, Bangladesh

^3^ University-Industry Cooperation Foundation, Suncheon National University, 255 Jungang-ro, Suncheon, Jeonnam 57922, South Korea

^4^ Damyang-gun Agricultural Technology Center, Damyang 57365, Korea

* **Correspondence:** htkim@sunchon.ac.kr (H.-T.K.), Tel.: +82617503242; nis@sunchon.ac.kr (I.-S.N.), Tel.: +82-617-503-249 (I.-S.N.); Fax: +82-617-505-389 (H.-T.K. & I.-S.N.)

^‡^ These authors contributed equally to this work.


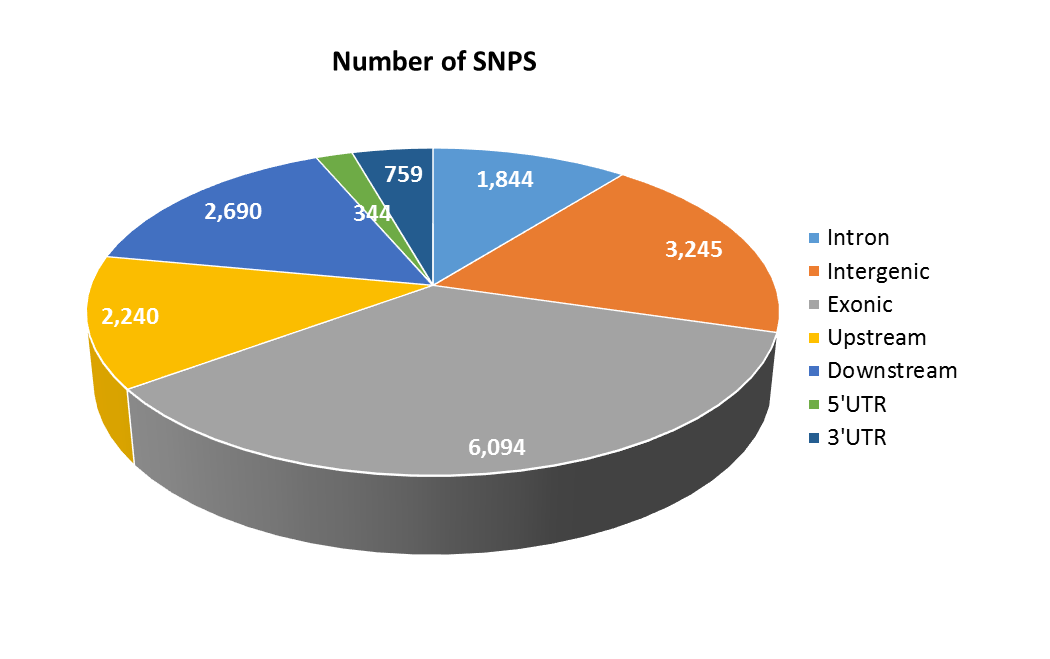


**Figure S1.** The functional distribution of SNPs identified from ddRAD-sequencing.


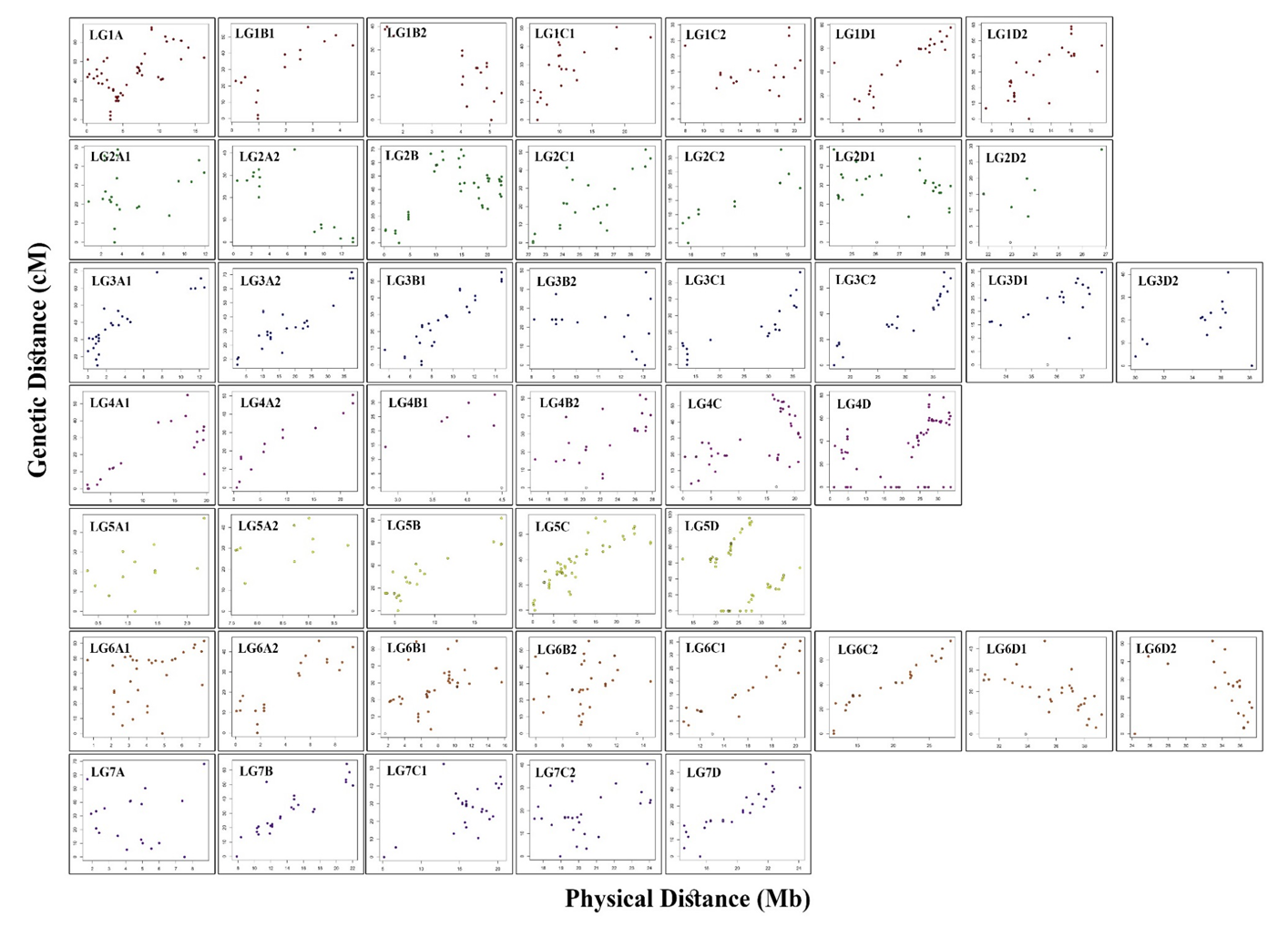


**Figure S2.** Marey maps showing the alignment of the genetic positions of the SNPs on the constructed genetic linkage map with regards to the physical positions (Mb) of the SNP associate sequence tags on the *F. vesca* genome (v4.0.a1).

**Table S1.** Statistical summary of barcodes, raw reads, mapped reads and reference genome alignment rate of each strawberry genotypes sequenced by ddRAD-sequencing technique.

| **SL** | **Samples** | **Barcodes** | **Raw reads** | **Mapped to reference genome** | | | |
| --- | --- | --- | --- | --- | --- | --- | --- |
|  |  |  |  | **Forward reads** | **Reverse reads** | **Total** | **Alignment rate** |
| 1 | Maehyang (M)-01 | TAAGGCGA | 1296564 | 931300 | 123449 | 1054749 | 81.35% |
| 2 | Maehyang (M)-02 | CGTACTAG | 1045339 | 746622 | 102425 | 849047 | 81.22% |
| 3 | Maehyang (M)-03 | AGGCAGAA | 1083038 | 785758 | 102730 | 888488 | 82.04% |
| 4 | Albion (A)-01 | CTCTCTAC | 1131230 | 792616 | 118184 | 910800 | 80.51% |
| 5 | Albion (A)-02 | CAGAGAGG | 1115965 | 784110 | 113900 | 898010 | 80.47% |
| 6 | Albion (A)-03 | TAAGGCGA | 1286735 | 914084 | 126429 | 1040513 | 80.86% |
| 7 | M x A-01 | CGTACTAG | 1399154 | 964924 | 144197 | 1109121 | 79.27% |
| 8 | M x A-02 | AGGCAGAA | 1365105 | 923088 | 146955 | 1070043 | 78.39% |
| 9 | M x A-03 | TCCTGAGC | 1135981 | 759714 | 125959 | 885673 | 77.97% |
| 10 | M x A-04 | GGACTCCT | 1278302 | 866232 | 137154 | 1003386 | 78.49% |
| 11 | M x A-05 | TAGGCATG | 1204499 | 816940 | 127757 | 944697 | 78.43% |
| 12 | M x A-06 | CTCTCTAC | 1284892 | 839992 | 146002 | 985994 | 76.74% |
| 13 | M x A-07 | CAGAGAGG | 1246873 | 861334 | 135847 | 997181 | 79.97% |
| 14 | M x A-08 | TAAGGCGA | 1409763 | 944780 | 152530 | 1097310 | 77.84% |
| 15 | M x A-09 | CGTACTAG | 1486207 | 1043380 | 152437 | 1195817 | 80.46% |
| 16 | M x A-10 | AGGCAGAA | 1292408 | 878918 | 138869 | 1017787 | 78.75% |
| 17 | M x A-11 | TCCTGAGC | 1252033 | 888764 | 122471 | 1011235 | 80.77% |
| 18 | M x A-12 | GGACTCCT | 1333430 | 917166 | 138279 | 1055445 | 79.15% |
| 19 | M x A-13 | TAGGCATG | 1240386 | 857580 | 128904 | 986484 | 79.53% |
| 20 | M x A-14 | CTCTCTAC | 1380659 | 921706 | 152854 | 1074560 | 77.83% |
| 21 | M x A-15 | CAGAGAGG | 1378650 | 942832 | 146955 | 1089787 | 79.05% |
| 22 | M x A-16 | TAAGGCGA | 1418770 | 1001496 | 139602 | 1141098 | 80.43% |
| 23 | M x A-17 | CGTACTAG | 1301830 | 941410 | 127348 | 1068758 | 82.10% |
| 24 | M x A-18 | AGGCAGAA | 1216844 | 861106 | 123051 | 984157 | 80.88% |
| 25 | M x A-19 | TCCTGAGC | 1081472 | 766062 | 93968 | 860030 | 79.52% |
| 26 | M x A-20 | GGACTCCT | 1246082 | 890682 | 125055 | 1015737 | 81.51% |
| 27 | M x A-21 | TAGGCATG | 1195963 | 851786 | 119267 | 971053 | 81.19% |
| 28 | M x A-22 | CTCTCTAC | 1255280 | 874026 | 129551 | 1003577 | 79.95% |
| 29 | M x A-23 | CAGAGAGG | 1297045 | 884038 | 147307 | 1031345 | 79.51% |
| 30 | M x A-24 | GCTACGCT | 1348010 | 968484 | 129163 | 1097647 | 81.43% |
| 31 | M x A-25 | CGAGGCTG | 1050707 | 755438 | 99849 | 855287 | 81.40% |
| 32 | M x A-26 | AAGAGGCA | 1209866 | 862626 | 126204 | 988830 | 81.73% |
| 33 | M x A-27 | GTAGAGGA | 1218448 | 853802 | 125801 | 979603 | 80.40% |
| 34 | M x A-28 | CGCGATAT | 966092 | 681654 | 103180 | 784834 | 81.24% |
| 35 | M x A-29 | CACGCGAG | 1017447 | 720732 | 109338 | 830070 | 81.58% |
| 36 | M x A-30 | ACGTATCA | 1080462 | 731678 | 114474 | 846152 | 78.31% |
| 37 | M x A-31 | CTACTATG | 1344287 | 944936 | 138986 | 1083922 | 80.63% |
| 38 | M x A-32 | GCTACGCT | 1384617 | 989200 | 137402 | 1126602 | 81.37% |
| 39 | M x A-33 | CGAGGCTG | 1282480 | 932838 | 120715 | 1053553 | 82.15% |
| 40 | M x A-34 | AAGAGGCA | 1131816 | 812638 | 114184 | 926822 | 81.89% |
| 41 | M x A-35 | GTAGAGGA | 201946 | 141942 | 24302 | 166244 | 82.32% |
| 42 | M x A-36 | CGCGATAT | 1232558 | 851588 | 127166 | 978754 | 79.41% |
| 43 | M x A-37 | CACGCGAG | 1024516 | 705460 | 108559 | 814019 | 79.45% |
| 44 | M x A-38 | ACGTATCA | 1239366 | 834184 | 139468 | 973652 | 78.56% |
| 45 | M x A-39 | CTACTATG | 1370492 | 957850 | 144997 | 1102847 | 80.47% |
| 46 | M x A-40 | GCTACGCT | 1497566 | 1035330 | 159659 | 1194989 | 79.80% |
| 47 | M x A-41 | CGAGGCTG | 1124430 | 798362 | 112079 | 910441 | 80.97% |
| 48 | M x A-42 | AAGAGGCA | 1417391 | 1025936 | 136210 | 1162146 | 81.99% |
| 49 | M x A-43 | GTAGAGGA | 1087215 | 748700 | 121605 | 870305 | 80.05% |
| 50 | M x A-44 | CGCGATAT | 1175163 | 845916 | 113883 | 959799 | 81.67% |

**Table S2-TableS6.** [available in separate spreadsheets online]

**Table S7.** List of selected genes within runner production related QTLs with potential role in vegetative vs reproductive differentiation of shoot apical meristems in strawberry.

| **Gene ID^x^** | **Description** | **Arabidopsis hit** | **QTL** | **Putative Role** | **Ref** |
| --- | --- | --- | --- | --- | --- |
| FvH4_5g17270 | WUSCHEL related homeobox 1 | AT3G18010 | qRU-4D | Specifying stem cell fate | ^1^ |
| FvH4_4g20680 | AGAMOUS-like 71 | AT5G51870 | qRU-4D | Termination of stem cell maintenance | ^2^ |
| FvH4_2g19210 | CLAVATA3/ESR (CLE)-related protein TDIF-like |  | qRU-4C | Signaling of cell fate decisions | ^3^ |
| FvH4_1g17150; FvH4_1g17160; FvH4_1g17170 | Subtilisin-like protease | AT5G67360 | qRU-1D2 | Shoot meristem differentiation | ^4^ |
| FvH4_5g32370; FvH4_5g32380 | Tetratricopeptide repeat (TPR)-like superfamily protein | AT1G71490 | qRU-5D | Directional cell division and maintenance of meristem cell organization | ^5^ |
| FvH4_1g17090; FvH4_1g17100 |  | AT5G43120 | qRU-1D2 |  |  |
| FvH4_1g17100 |  | AT4G37460 | qRU-1D2 |  |  |
| FvH4_4g21400 |  | AT3G06430 | qRU-4D |  |  |
| FvH4_2g19310 |  | AT5G48850 | qRU-4C |  |  |
| FvH4_2g19970 |  | AT4G28740 | qRU-4C |  |  |
| FvH4_2g20050 |  | AT2G33680 | qRU-4C |  |  |
| FvH4_2g20260 |  | AT1G04130 | qRU-4C |  |  |
| FvH4_5g17170 |  | AT4G02750 | qRU-5C |  |  |
| FvH4_2g30660 |  | AT5G24830 | qRU-2D2 |  |  |
| FvH4_2g30790 |  | AT4G33170 | qRU-2D2 |  |  |
| FvH4_4g19750 | Phytochrome B-like | AT2G18790 | qRU-4D | photoperiodic control of flowering | ^6^ |
| FvH4_2g19790 | Phytochrome interacting factor 3-like 5 | AT2G20180 | qRU-4C |  |  |
| FvH4_4g20210 | Flowering time control protein FCA-like isoform X1 | AT4G16280 | qRU-4D | Flowering time control | - |
| FvH4_2g19520 | C2H2-like zinc finger protein KNUCKLES-like | AT5G48890 | qRU-4C |  | - |
| FvH4_2g30770 | Flowering-promoting factor 1-like (FPF1) | AT5G24860 | qRU-2D2 |  | - |
| FvH4_5g17130 | Mother of FT (FLOWERING LOCUS T) and TF1 (TERMINAL FLOWER 1); PEBP | - | qRU-5C |  | ^7^ |
| FvH4_5g18150; FvH4_5g18160 | Protein argonaute 1; AGO1 | AT1G48410 | qRU-5C |  | ^8,9^ |
| FvH4_5g16850 | AP2/B3-like transcriptional factor; VRN1 | AT3G18990 | qRU-5C | Vernalization and flowering time control | ^10^ |
| FvH4_4g19970 | Putative calmodulin-like protein 2 | AT5G49480 | qRU-4D | regulator of flowering | ^11^ |
| FvH4_5g16470 | Calmodulin binding protein-like | AT5G62570 | qRU-5C |  |  |
| FvH4_5g17810 | Calmodulin 7; CAM7 | AT3G43810 | qRU-5C |  |  |
| FvH4_2g30990;  FvH4_2g31000;  FvH4_2g31010 | Cytokinin dehydrogenase 7 | AT5G21482 | qRU-2D2 | CYTOKININ–AUXIN mediated MERISTEM differentiation | ^12^ |
| FvH4_2g19860 | Auxin response factor 17-like | AT1G77850 | qRU-4C |  |  |
| FvH4_5g17310 | Auxin efflux carrier family protein | AT1G73590 | qRU-5C |  |  |
| FvH4_5g18130 | NAC domain-containing protein 86 | AT3G17730 | qRU-5C |  | ^13^ |
| FvH4_5g17670 | Homeobox protein knotted-1-like; KNOXI | AT1G14760 | qRU-5C |  | ^14^ |
| FvH4_5g17840 | SHOOT GRAVITROPISM 5-like | AT1G68130 | qRU-5C | gravitropism  and meristematic competence | ^15^ |
| FvH4_4g20730 | Gibberellin 2-beta-dioxygenase 2-like | AT1G02400 | qRU-4D | Flowering regulatory factor | ^16^ |
| FvH4_4g20660 | Translation elongation factor EF1B |  | qRU-4D | Meristem Stability and Organogenesis | ^17^ |
| FvH4_4g19950 | Eukaryotic translation initiation factor 3 subunit A-like; EIF3A | AT4G11420 | qRU-4D |  |  |
| FvH4_5g17000 | Translation elongation factor EF1A | AT1G18070 | qRU-5C |  |  |

**Supplementary references:**

1. Zuo, J., Niu, Q. W., Frugis, G. & Chua, N. H. The WUSCHEL gene promotes vegetative-to-embryonic transition in Arabidopsis. *Plant J.* **30,** 349–359 (2002).

2. Lenhard, M., Bohnert, A., Jürgens, G. & Laux, T. Termination of stem cell maintenance in Arabidopsis floral meristems by interactions between WUSCHEL and AGAMOUS. *Cell* **105,** 805–814 (2001).

3. Fletcher, J. C., Brand, U., Running, M. P., Simon, R. & Meyerowitz, E. M. Signaling of cell fate decisions by CLAVATA3 in Arabidopsis shoot meristems. *Science (80-. ).* **283,** 1911–1914 (1999).

4. Liu, J. X., Srivastava, R. & Howell, S. Overexpression of an arabidopsis gene encoding a subtilase (AtSBT5.4) produces a clavata-like phenotype. *Planta* **230,** 687–697 (2009).

5. Sotta, N., Shantikumar, L., Sakamoto, T., Matsunaga, S. & Fujiwara, T. TPR5 is involved in directional cell division and is essential for the maintenance of meristem cell organization in Arabidopsis thaliana. *J. Exp. Bot.* **67,** 2401–2411 (2016).

6. Izawa, T., Oikawa, T., Tokutomi, S., Okuno, K. & Shimamoto, K. Phytochromes confer the photoperiodic control of ¯owering in rice (a short-day plant). *Plant J.* **22,** 391–399 (2000).

7. An, H. *et al.* CONSTANS acts in the phloem to regulate a systemic signal that induces photoperiodic flowering of Arabidopsis. *Development* **131,** 3615–3626 (2004).

8. Kidner, C. A. & Martienssen, R. A. The role of ARGONAUTE1 (AGO1) in meristem formation and identity. *Dev. Biol.* **280,** 504–517 (2005).

9. Zhang, Z. & Zhang, X. Argonautes compete for miR165/166 to regulate shoot apical meristem development. *Curr. Opin. Plant Biol.* **15,** 652–658 (2012).

10. Levy, Y. Y., Mesnage, S., Mylne, J. S., Gendall, A. R. & Dean, C. Multiple roles of Arabidopsis VRN1 in vernalization and flowering time control. *Science (80-. ).* **297,** 243–246 (2002).

11. Liang, S., Wang, X., Lü, Y. & Feldman, L. J. Mediation of flowering by a calmodulin-dependent protein kinase. *Sci. China Ser. C Life Sci.* **44,** 506–512 (2001).

12. Su, Y. H., Liu, Y. B. & Zhang, X. S. Auxin-cytokinin interaction regulates meristem development. *Mol. Plant* **4,** 616–625 (2011).

13. Olsen, A. N., Ernst, H. A., Leggio, L. Lo & Skriver, K. NAC transcription factors: structurally distinct, functionally diverse. *Trends Plant Sci.* **10,** 79–87 (2005).

14. Yanai, O. *et al.* Arabidopsis KNOXI proteins activate cytokinin biosynthesis. *Curr. Biol.* **15,** 1566–1571 (2005).

15. Herranz, R. & Medina, F. J. Cell proliferation and plant development under novel altered gravity environments. *Plant Biol.* **16,** 23–30 (2014).

16. Mutasa-Gottgens, E. & Hedden, P. Gibberellin as a factor in floral regulatory networks. *J. Exp. Bot.* **60,** 1979–1989 (2009).

17. Zhou, F., Roy, B., Dunlap, J. R., Enganti, R. & von Arnim, A. G. Translational control of Arabidopsis meristem stability and organogenesis by the eukaryotic translation factor eIF3h. *PLoS One* **9,** e95396 (2014).

==()==
